# Knowledge and Attitudes of Nursing Mothers As Potential Determinants of Effective Breastfeeding

**DOI:** 10.7759/cureus.89926

**Published:** 2025-08-12

**Authors:** Aebel Raju, Athira Thomas, Biran Antony, Parvathi Vannathan Kandiyil, Ajithkumar V T., Sindhu T G.

**Affiliations:** 1 Department of Pediatrics, Government Medical College, Kozhikode, Kozhikode, IND

**Keywords:** breastfeeding woman, latch, low-risk neonates, postnatal weight gain, practice patterns, social practice

## Abstract

Background: Breastfeeding is crucial for infant nutrition and health. Knowledge, attitude, and practices of mothers significantly influence breastfeeding outcomes. This study evaluated mothers' knowledge and attitude toward breastfeeding and its correlation with effective breastfeeding, using the Latch, Audible swallowing, Type of nipple, Comfort, Hold (LATCH) score as a standard assessment.

Methods: This prospective study was conducted among 310 mothers admitted to wards at a tertiary center of healthcare from November 2021 to August 2022. A structured questionnaire assessed maternal knowledge and attitude. The effectiveness of breastfeeding was evaluated using the LATCH score, and infants were followed up at six weeks postpartum to assess weight gain. Statistical analyses involved descriptive statistics and Spearman's correlation. A p value of ≤0.05 was considered statistically significant.

Results: Of the 310 mothers, 88% initiated breastfeeding within the first hour after birth. The median knowledge score was 13. Mothers demonstrated overall positive attitudes toward breastfeeding, scoring high on a five-point Likert scale. The median LATCH score was 9. A statistically significant positive correlation was found between maternal knowledge scores and LATCH scores (Spearman’s rho = 0.435, CI = 0.432-0.468, p = 0.044). High LATCH scores correlated strongly with infant weight gain at six weeks (Spearman’s rho = 0.825, CI = 0.765-0.871, p = 0.001).

Conclusions: This study highlights the critical role of maternal knowledge and attitude in promoting successful breastfeeding. Mothers with a better understanding of breastfeeding practices demonstrated higher LATCH scores. A higher proportion of mothers who initiated breastfeeding within the first hour still faced challenges in applying correct techniques, requiring extra practical training, family support, and structured follow-up care to sustain breastfeeding effectively. By combining knowledge with accessible, real-world support, we can empower more mothers to breastfeed effectively, ultimately improving early childhood health and development.

## Introduction

Breastfeeding, recognized by the World Health Organization (WHO) as the optimal feeding method for infants, significantly reduces morbidity and mortality while promoting optimal growth and development. Exclusive breastfeeding (EBF) is advised until six months of age, extending up to two years or beyond, alongside complementary feeding. Despite global advocacy, breastfeeding practices often fall short due to limited maternal knowledge, misconceptions, and cultural attitudes [1].

There are regional differences in exclusive breastfeeding practices. The global weighted prevalence was 51.9% for early initiation of breastfeeding and 45.7% for EBF under six months [2]. In contrast, the national average is marked at 55% [3].

This study assesses the relationship between maternal knowledge and attitudes toward breastfeeding and its impact on breastfeeding effectiveness, measured using the Latch, Audible swallowing, Type of nipple, Comfort, Hold (LATCH) scoring system, in a tertiary care setting in Kozhikode, Kerala, India. The LATCH score is widely recognized as a crucial predictor of breastfeeding efficacy and the need for support among new mothers. Studies have demonstrated that a LATCH score greater than 6 at discharge is highly sensitive and specific for predicting EBF at six weeks postpartum [4]. Identifying the problems with breastfeeding early through the LATCH score can help in early interventions, which could bring out longer term benefits [5].

## Materials and methods

Study design

This prospective cohort study was conducted from November 2021 to August 2022 among 310 mothers admitted to the antenatal and postnatal wards of Government Medical College, Kozhikode. Objectives of this study are: to assess maternal knowledge and attitudes toward breastfeeding, correlate knowledge/attitude scores with LATCH scores, and evaluate the association between LATCH scores and infant weight gain at six weeks. Maternal knowledge and attitudes toward breastfeeding were assessed using a structured questionnaire. Breastfeeding effectiveness was objectively evaluated using the LATCH scoring tool, and infant weight gain was recorded during a follow-up visit at six weeks postpartum. Descriptive statistics and Spearman’s correlation were used for analysis, with a p value of ≤0.05 considered statistically significant.

Participants

**Figure 1:**
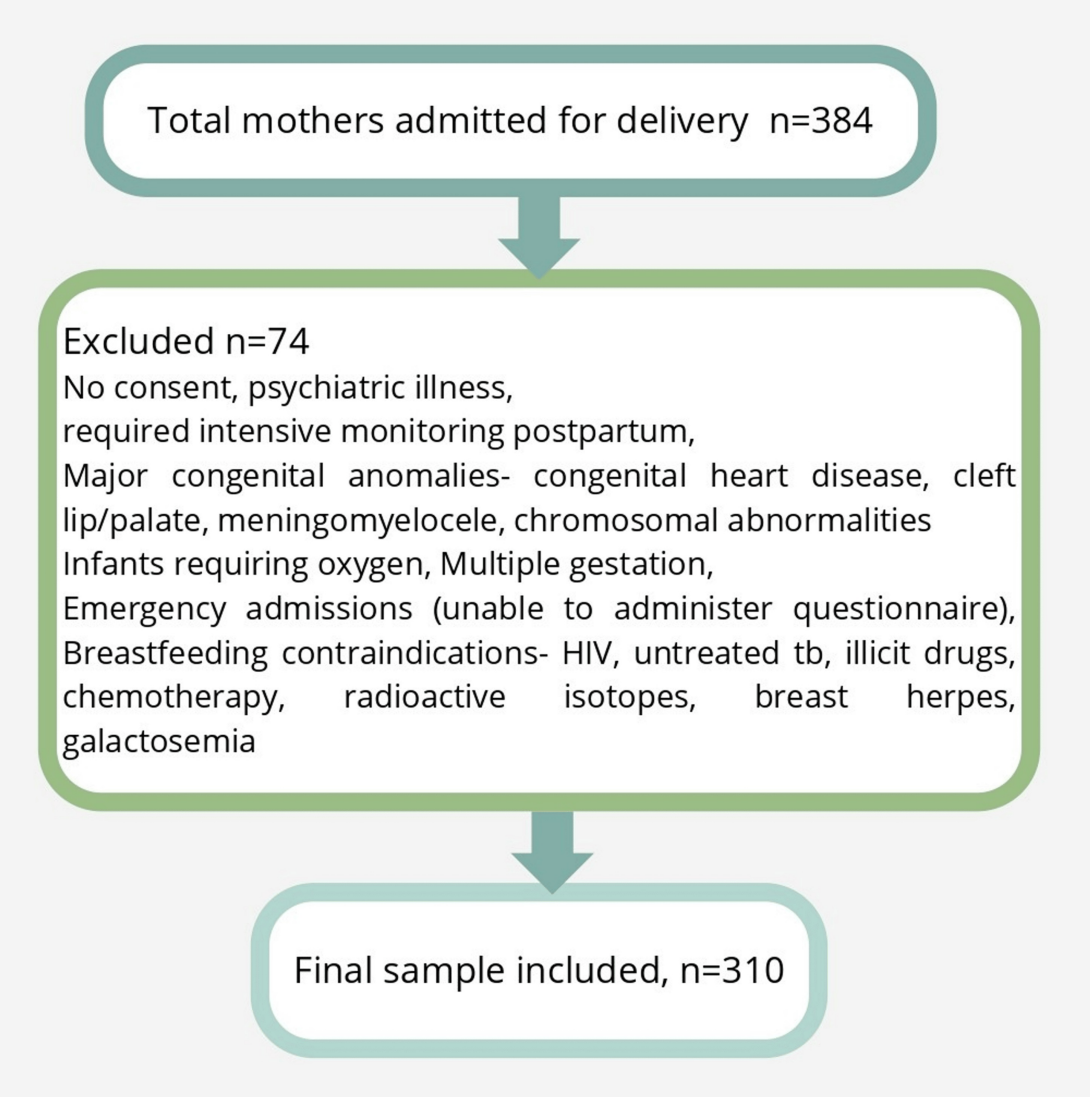
Flowchart showing participant selection process for the study A total of 384 mothers admitted for delivery were assessed for eligibility. After applying exclusion criteria, including lack of consent, psychiatric illness, need for intensive postnatal monitoring, presence of major congenital anomalies in the infant, multiple gestations, emergency admissions, and medical contraindications to breastfeeding, a total of 310 mothers were included in the final analysis

The study included 310 mothers who were admitted for delivery at a gestational age of 37 weeks or more during the study period at a tertiary care center by universal sampling. Mothers were excluded if they did not provide informed consent, had diagnosed psychiatric illnesses, or required intensive monitoring in the postnatal period. Additionally, mothers whose babies had major congenital anomalies, such as congenital heart disease, cleft lip or palate, meningomyelocele, and chromosomal abnormalities, or who required oxygen support, were excluded. Other exclusion criteria included multiple gestations, emergency admissions where the awareness questionnaire could not be administered, and medical contraindications to breastfeeding. These contraindications included maternal HIV infection, active untreated tuberculosis, use of illicit drugs, administration of contraindicated medications (e.g., chemotherapy and radioactive isotopes), active herpes simplex lesions on the breast, and infants diagnosed with galactosemia (Figure 1).

Data collection

Maternal knowledge was assessed through a 15-item questionnaire covering topics such as the benefits of breastfeeding, feeding frequency, positioning, duration, and recognition of infant hunger cues. This was developed based on multiple literature reviews and department faculty peer review. Each correct response was scored as one point, with a higher cumulative score reflecting better knowledge. Based on a maximum possible knowledge score of 15, the participants' knowledge levels were classified into three categories: Good knowledge was defined as a score between 12 and 15, indicating a strong understanding of breastfeeding practices likely to support optimal outcomes. Fair knowledge was assigned to scores between 8 and 11, reflecting moderate awareness that may benefit from further education and support. Poor knowledge was considered for scores below 8, indicating limited or incorrect understanding and a higher risk of ineffective breastfeeding practices. The questionnaire was piloted among 20 mothers (excluded from the main study) to assess clarity and relevance. Cronbach’s alpha confirmed internal consistency. Feedback refined ambiguous terms.

Attitude toward breastfeeding was evaluated using a set of statements rated on a five-point Likert scale ranging from “strongly disagree” to “strongly agree.” The attitude score reflected the level of positivity in the mother’s perception of breastfeeding, with higher scores indicating a more favorable attitude. Each item was scored from 1 (Strongly Disagree) to 5 (Strongly Agree), with negatively worded items reverse-scored. The total score ranged from 16 to 80, and a score of ≥64 (80%) was classified as indicating a positive attitude toward breastfeeding.

Breastfeeding practices were directly observed and scored using the validated LATCH tool, which assesses five components: Latch, Audible swallowing, Type of nipple, Comfort of the mother, and Hold (positioning). Each component was rated from 0 to 2, with a maximum total score of 10 [4]. LATCH assessments were conducted by four trained nurses certified in the WHO lactation guidelines. Interrater reliability (Cohen's κ) was established via joint scoring of 30 pilot cases. Discrepancies were resolved through consensus. Observations were performed within the first 48 hours postpartum by trained, blinded personnel to ensure consistency. The effectiveness of breastfeeding was objectively assessed based on infant weight gain at six weeks postpartum, measured using calibrated weighing scales, and compared with birth weight. Measurements were taken nude, prefeed, by trained nurses blinded to maternal LATCH scores. Weight gain was calculated as the difference (grams) from birth.

Data were analyzed using both descriptive and inferential statistics. Correlations between knowledge scores, attitude scores, LATCH scores, and infant weight gain were determined using Spearman’s rank correlation coefficient. A p value of ≤0.05 was considered statistically significant. We hypothesized that higher maternal knowledge and a more positive attitude toward breastfeeding would be positively correlated with LATCH scores and infant weight gain at six weeks postpartum.

Statistical analysis

The statistical analysis was performed using IBM Statistical Package for the Social Sciences Statistics (version 29, IBM Corporation, Armonk, NY).

## Results

A total of 310 postpartum mothers meeting the inclusion criteria were enrolled in the study. The demographic characteristics of the participants are presented in Table 1. The majority were aged 21-25 years (40%) and primigravida (60%), and had a normal BMI (60%). Most deliveries were normal vaginal (80%).

**Table 1:** Demographic profile of study participants (n = 310) BMI: body mass index

Characteristic	Category	Frequency (n)	Percentage (%)
Age (years)	≤20	62	20
	21-25	124	40
	26-30	78	25.2
	>30	46	14.8
Education of mother	Primary school certificate	31	10
	Middle school certificate	31	10
	High school certificate	124	40
	Intermediate/diploma	62	20
	Graduate or higher	62	20
Occupation of mother	Unemployed	217	70
	Skilled workers	47	15.2
	Elementary occupations	31	10
	Professionals/technicians	15	4.8
Monthly family income (₹)	≤6,174	124	40
	6,175-18,496	93	30
	18,497-30,830	62	20
	>30,830	31	10
BMI	≤18.5 (underweight)	62	20
	18.5-24.9 (normal)	186	60
	25-29.9 (overweight)	46	14.8
	≥30 (obese)	16	5.2
Parity	Primigravida	186	60
	Multigravida (G2-G3)	93	30
	Grand multipara (≥G4)	31	10
Type of delivery	Normal vaginal delivery	248	80
	Cesarean section	62	20

**Figure 2:**
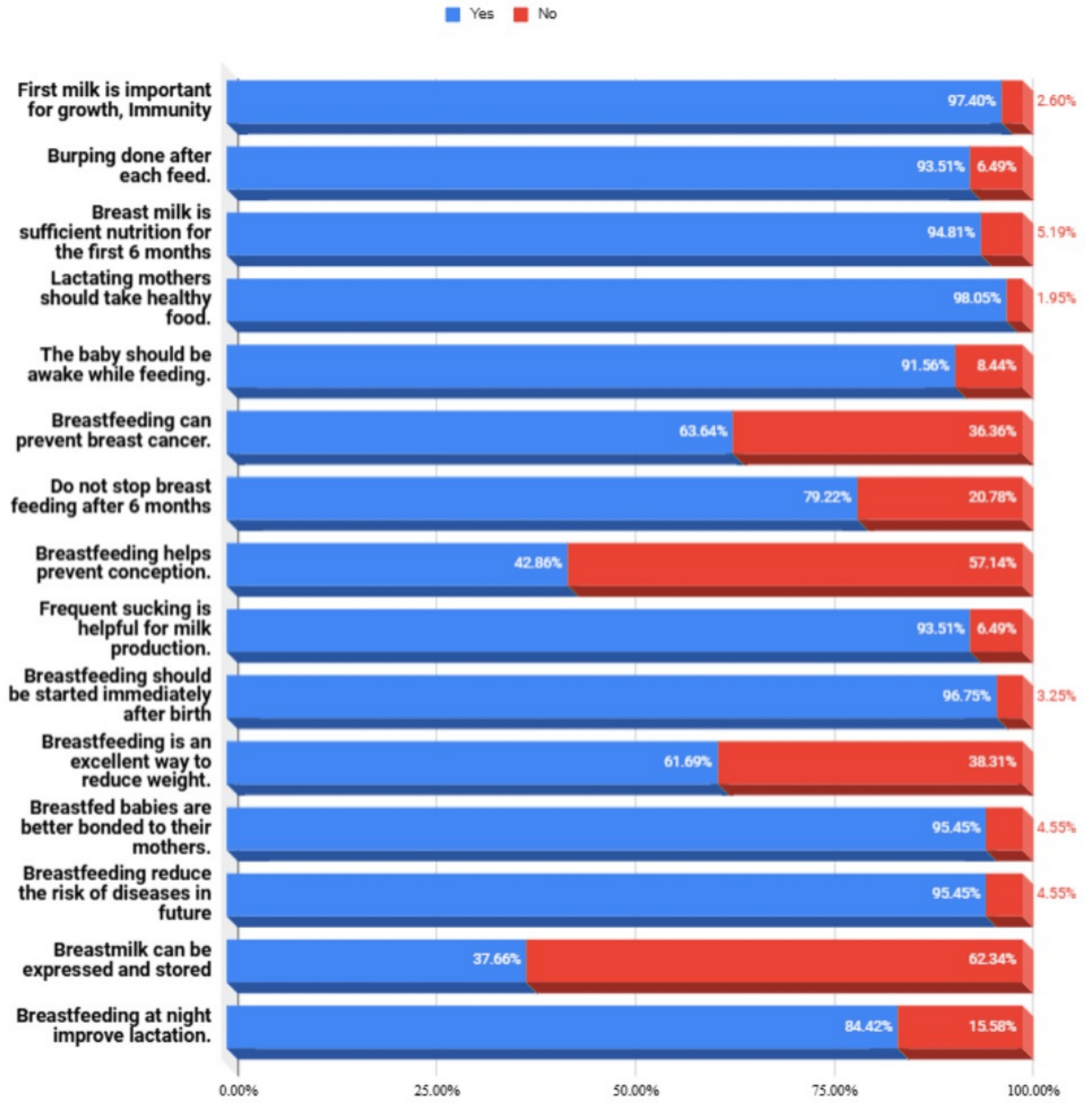
Distribution of responses to 15 knowledge-based questions assessing mothers' understanding of breastfeeding Each item was scored as either "Yes" (correct) or "No" (incorrect), with percentages shown for each response

Early initiation of breastfeeding within the first hour postpartum was observed in 88% of mothers, significantly higher than the reported Indian national average (55%). The median maternal knowledge score was 13 (range: 6-15). Based on predefined categories, 74.9% of mothers demonstrated good knowledge, 23.1% had fair knowledge, and only 2% had poor knowledge. Key areas of strong awareness included the benefits of colostrum (92%), immunological advantages of breastfeeding (89%), and recognition of infant hunger cues (85%) (Figure 2). Maternal knowledge scores showed no significant correlation with education level (Spearman’s ρ = 0.09, p = 0.12) or employment status (p = 0.67, analysis of variance).

**Figure 3:**
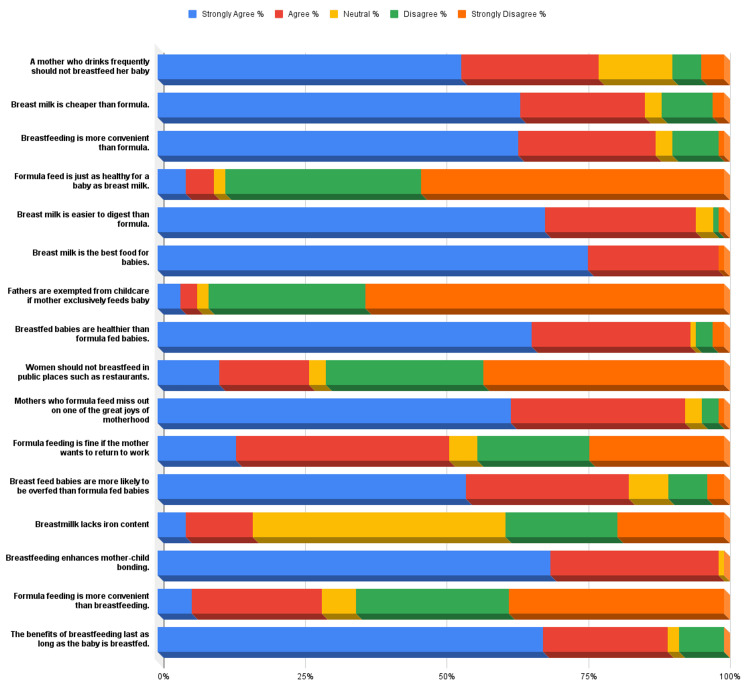
Attitude chart This horizontal stacked bar chart presents mothers’ responses to 16 attitude-based statements related to breastfeeding, using a five-point Likert scale: Strongly Agree, Agree, Neutral, Disagree, and Strongly Disagree

Attitudinal assessment revealed that 87% of mothers strongly agreed or agreed that breastfeeding promotes mother-infant bonding. Similarly, 86% endorsed breastfeeding as superior to formula feeding, while 88% acknowledged its long-term health benefits. Less than 10% of participants expressed neutral or negative views, indicating an overall positive attitude toward breastfeeding (Figure 3).

**Figure 4:**
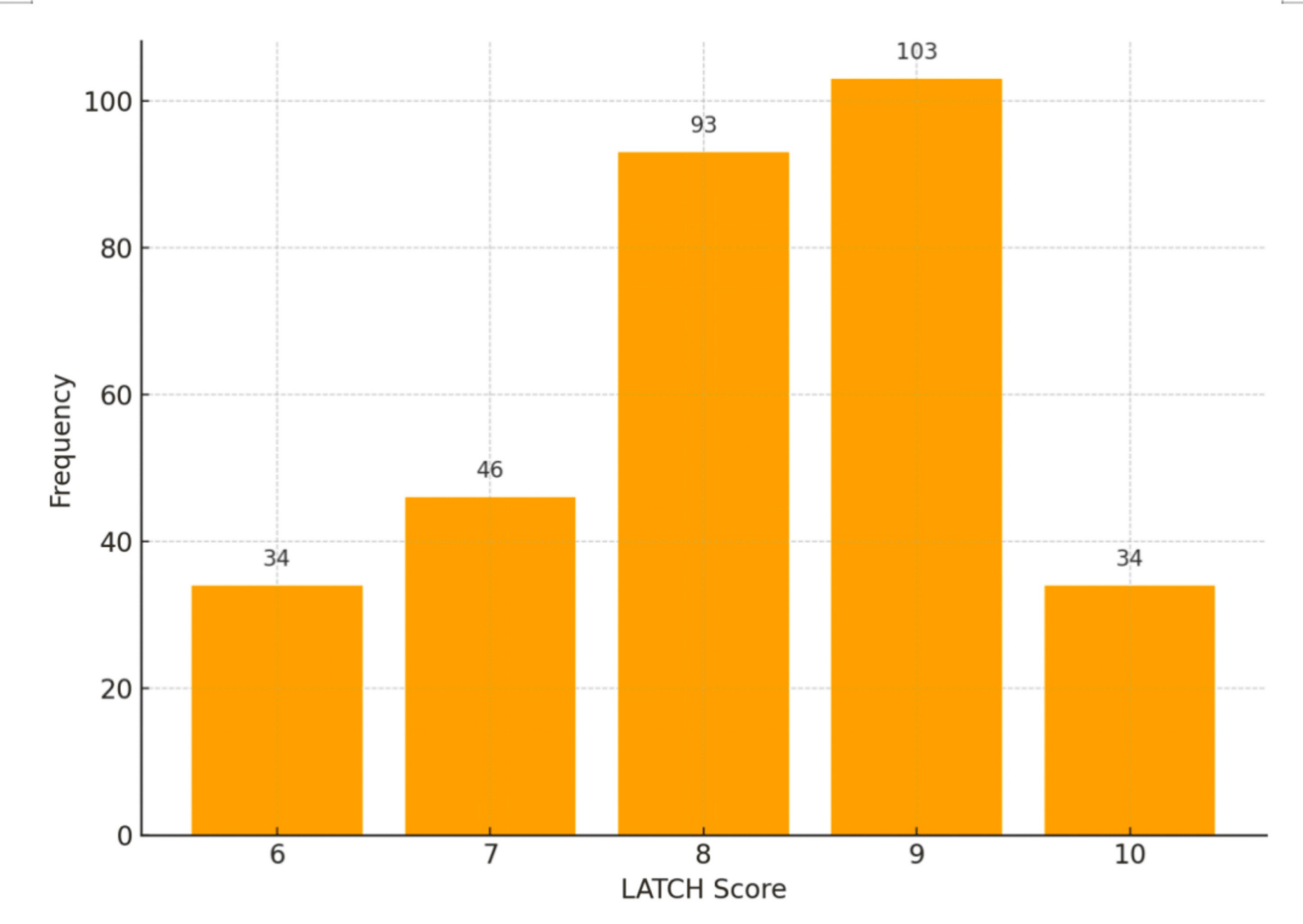
Bar chart illustrating the frequency distribution of LATCH scores recorded during the first breastfeeding session after delivery The LATCH tool, a validated instrument for assessing breastfeeding effectiveness, ranges from 0 to 10 LATCH: Latch, Audible swallowing, Type of nipple, Comfort, Hold

The median LATCH score was 9 (range: 6-10), with 78% of mothers scoring ≥8, reflecting effective breastfeeding techniques. A statistically significant positive correlation was observed between maternal knowledge scores and LATCH scores (Spearman’s ρ = 0.435, 95% CI = 0.30-0.55; *p* = 0.044). Furthermore, higher LATCH scores were associated with greater infant weight gain at six weeks postpartum (ρ = 0.825, 95% CI = 0.765-0.871; *p* = 0.001) (Figure 4).

## Discussion

In this prospective study involving 310 postpartum mothers, we observed a generally positive outlook toward breastfeeding, reflected in both high maternal knowledge and favorable attitudes. The effectiveness of breastfeeding was quantitatively assessed using the LATCH score, with a significant positive correlation noted between maternal knowledge and breastfeeding success (Spearman’s rho = 0.435, p = 0.044). Additionally, a strong association was found between LATCH scores and infant weight gain at six weeks postpartum (Rho = 0.825, p = 0.001), reinforcing the clinical importance of early breastfeeding competency in improving neonatal outcomes. These findings are supported by previous literature, which has demonstrated that higher LATCH scores correlate with improved infant growth and effective breastfeeding practices [5-7].

Kul Uçtu and Özerdoğan conducted a randomized controlled trial examining the teach-back method's impact on breastfeeding self-efficacy. Their study reported significant improvement in LATCH scores (from 7.73 to 9.95, p < 0.001) following structured maternal education. Similarly, our study affirms that enhanced maternal knowledge positively influences LATCH scores and breastfeeding performance [8].

Interestingly, our data showed no statistically significant relationship between maternal education or employment status and breastfeeding knowledge or LATCH score, diverging from earlier studies that linked higher education levels with greater breastfeeding success [9,10]. This finding may reflect the positive influence of universal public health initiatives in bridging educational disparities, especially in tribal and underserved communities. Despite a fair level of formal education, the lack of correlation underscores the distinction between general education and specific breastfeeding knowledge, as supported by prior studies [11]. This could highlight that targeted antenatal education programs focused specifically on lactation practices can remove the disparity in the feeding practices of even the unemployed and uneducated class of the maternal population.

Attitudinal responses were overwhelmingly positive in our cohort. Over 85% of mothers agreed or strongly agreed that breastfeeding promotes mother-infant bonding and provides superior health benefits compared to formula feeding. This positive outlook could serve as a strong foundation for structured educational interventions, such as the teach-back method, to enhance practical adherence in regions with variable health literacy, and this method could be utilized for spreading awareness among the community belonging to lower social and economic status [12].

However, positive attitudes did not always translate into effective breastfeeding techniques. Despite early initiation rates reaching 88%, higher than national averages, several mothers faced challenges in applying correct techniques. These barriers may include maternal fatigue, limited autonomy, and insufficient family support. Such discrepancies are consistent with findings from studies conducted in Ghana and Ethiopia, where favorable attitudes failed to ensure consistent EBF [13-15]. These results suggest that attitudes alone are insufficient and must be reinforced with hands-on skill development and ongoing support.

Consistent with global evidence, higher LATCH scores postpartum were predictive of successful EBF at six weeks [4,16,17]. Our center’s integration of routine LATCH assessments into postnatal care, in alignment with Baby-Friendly Hospital Initiative guidelines, has proven effective in identifying mothers requiring early lactation support. This structured approach not only improves lactation outcomes but also supports evidence-based best practices in neonatal care.

This study has several limitations. Being a single-center study, the generalizability of results to other populations or geographical settings may be limited. The use of universal sampling, recall bias, and social desirability bias. Although comprehensive, it lacks the methodological rigor of randomized sampling techniques, which would improve external validity. Furthermore, we did not control for potential confounding variables, such as family support systems or prior breastfeeding experience, both of which could significantly influence maternal confidence and breastfeeding outcomes. Identifying maternal challenges was not within the predefined objectives of our study, which focused on quantitative associations between knowledge, attitude, LATCH scores, and infant weight gain.

## Conclusions

This study highlights the critical role of maternal knowledge and attitude in promoting successful breastfeeding. Mothers with a better understanding of breastfeeding practices demonstrated higher LATCH scores, which correlated with more effective breastfeeding and greater infant weight gain by six weeks postpartum. Although a high proportion of mothers (88%) initiated breastfeeding within the first hour, well above the national average, many still faced challenges in applying correct techniques. This indicates that awareness alone is insufficient; mothers also require practical training, family support, and structured follow-up care to sustain breastfeeding effectively.

The LATCH scoring system proved to be a valuable tool for the early identification of mothers who may benefit from additional lactation support. To enhance breastfeeding outcomes, healthcare systems should invest in structured educational programs, hands-on skill development, and consistent postnatal counseling. Furthermore, supportive workplace policies, improved maternity care services, and community-driven awareness initiatives can collectively foster an environment conducive to successful breastfeeding. By combining knowledge with accessible, real-world support, we can empower more mothers to breastfeed effectively, ultimately improving early childhood health and development.
